# Role of Preoperative Abdominal MRI in Preventing Futile Laparotomy or Diagnostic Staging Laparoscopy in the Setting of Pancreatic Adenocarcinoma

**DOI:** 10.1002/wjs.70486

**Published:** 2026-07-08

**Authors:** Joshua O. Cerasuolo, Pablo E. Serrano, Noori Akhtar‐Danesh, Amer Alaref, David W. Savage, Joseph M. Caswell, Christian B. van der Pol

**Affiliations:** ^1^ Department of Health Research Methods, Evidence, and Impact Faculty of Health Sciences McMaster University Hamilton Ontario Canada; ^2^ ICES North Health Sciences North Research Institute Sudbury Ontario Canada; ^3^ Department of Surgery Faculty of Health Sciences McMaster University Hamilton Ontario Canada; ^4^ Juravinski Hospital and Cancer Center Hamilton Health Sciences Hamilton Ontario Canada; ^5^ ICES McMaster Faculty of Health Sciences McMaster University Hamilton Ontario Canada; ^6^ School of Nursing Faculty of Health Sciences McMaster University Hamilton Ontario Canada; ^7^ NOSM University Thunder Bay Ontario Canada; ^8^ Thunder Bay Regional Health Sciences Center Thunder Bay Ontario Canada; ^9^ Department of Medical Imaging Faculty of Health Sciences McMaster University Hamilton Ontario Canada

**Keywords:** futile laparotomy, magnetic resonance imaging, pancreatic ductal adenocarcinoma, pancreatic neoplasms, population, retrospective study

## Abstract

Among patients with pancreatic cancer deemed resectable on computed tomography (CT), approximately 41% have more advanced disease that is not detected by CT. Diagnostic staging laparoscopy is used preoperatively either routinely or selectively in patients with high‐risk features and may reduce unnecessary laparotomies by more than 20%. However, it remains an invasive procedure that requires general anesthesia and, when possible, should be avoided if metastatic disease is already evident on preoperative imaging. CT remains the standard imaging modality for staging pancreatic cancer and determining its resectability, but recent studies have demonstrated the added benefit of other radiological modalities in reducing unnecessary surgeries. Magnetic resonance imaging (MRI) is recommended for patients with high risk features to detect extra‐pancreatic metastases or for those with indeterminate liver lesions on CT. Accordingly, at a population‐level, we evaluated whether patients diagnosed with pancreatic adenocarcinoma and receiving preoperative abdominal MRI, in lieu of or combined with CT, had a lower likelihood of laparotomy and/or laparoscopy.

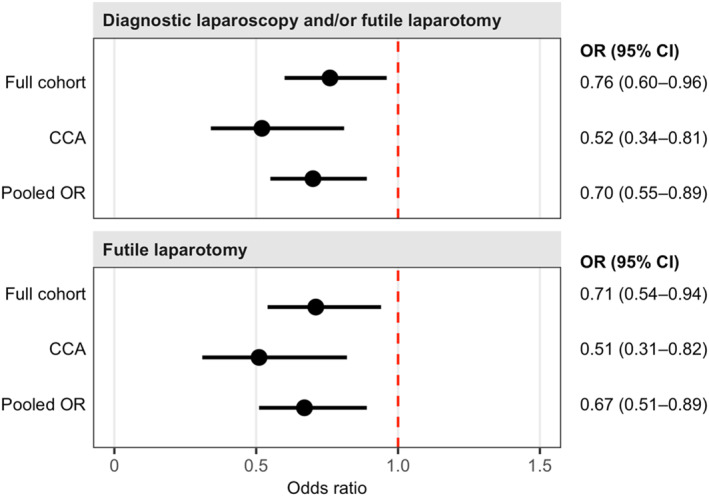

## 1 Introduction

Among patients with pancreatic cancer deemed resectable on computed tomography (CT), approximately 41% have more advanced disease that is not detected by CT [1]. Diagnostic staging laparoscopy is used preoperatively either routinely or selectively in patients with high‐risk features and may reduce unnecessary laparotomies by more than 20% [1]. However, it remains an invasive procedure that requires general anesthesia and, when possible, should be avoided if metastatic disease is already evident on preoperative imaging. CT remains the standard imaging modality for staging pancreatic cancer and determining its resectability, but recent studies have demonstrated the added benefit of other radiological modalities in reducing unnecessary surgeries [2, 3, 4, 5, 6]. Magnetic resonance imaging (MRI) is recommended for patients with high risk features to detect extra‐pancreatic metastases or for those with indeterminate liver lesions on CT [7]. Accordingly, at a population‐level, we evaluated whether patients diagnosed with pancreatic adenocarcinoma and receiving preoperative abdominal MRI, in lieu of or combined with CT, had a lower likelihood of laparotomy and/or laparoscopy.

## 2 Methods

Using a population‐based provincial cancer registry, we included all adults (≥ 18 years) diagnosed with pancreatic adenocarcinoma from January 2011 to December 2023 (International Classification of Diseases for Oncology, third edition codes: C25.0, C25.1, C25.2, C25.3, C25.7, C25.8, and C25.9). Surgical and radiological procedures were ascertained from physician billing records in the Ontario Health Insurance Plan (OHIP) database. Stage was obtained from the Ontario Cancer Registry (OCR) as *de novo* TNM stage and categorized as stage I, II, III, or unknown/not staged. Administration of neoadjuvant therapy was extracted from cancer clinic data. We excluded patients who were not living in Ontario nor eligible for provincial health insurance (*n* = 16), did not undergo preoperative abdominal CT or MRI (*n* = 36), or were diagnosed with *de novo* stage IV cancer as this is a contraindication for resection (*n* = 512). Finally, atypical cases with an interval greater than 365 days between diagnosis and first surgical intervention were excluded (*n* = 94). All datasets used in this study were linked using unique encoded identifiers and analyzed at ICES.

The primary exposure was receipt of preoperative abdominal MRI. The primary clinical endpoint was a composite of futile laparotomy and/or diagnostic staging laparoscopy, defined as either: (1) laparotomy or laparoscopy performed without subsequent resection, or (2) biliary or gastrointestinal bypass without resection. Patients who subsequently underwent resection were classified as *not* having experienced futile laparotomy or diagnostic staging laparoscopy. In Ontario, Canada, planned bypass procedures without resection are uncommon because of the widespread availability of biliary and gastrointestinal stenting, performed either endoscopically or by interventional radiology. Futile laparotomy was evaluated both as an independent outcome and in combination with diagnostic staging laparoscopy, given that laparoscopy remains an invasive surgical procedure that may delay the administration of systemic therapy and consume finite healthcare resources, outcomes that may potentially be mitigated by more definitive preoperative imaging such as MRI.

To address confounding by indication and achieve balance in baseline characteristics between patients who did versus did not undergo preoperative abdominal MRI, we used a propensity score‐weighted design with inverse probability of treatment weights [8, 9]. Weighted generalized linear models with a binomial distribution and logit link were used to estimate odds ratios (ORs) for the association between preoperative abdominal MRI and the occurrence of futile laparotomy or diagnostic staging laparoscopy. Selected based on data availability, the exposure groups were balanced on age, sex, geographic remoteness [10], time from diagnosis to surgery (in days), neighbourhood income quintile, Charlson comorbidity index, neoadjuvant radiation, neoadjuvant chemotherapy, and *de novo* TNM stage. Because approximately 40% of participants had missing or unknown stage, we performed three complimentary analyses to assess the robustness of our findings: (1) a full cohort analysis retaining missing or unknown stage as a separate category; (2) a complete‐case analysis excluding patients with missing or unknown stage; and (3) propensity score analyses across 50 multiply imputed datasets with cancer stage imputed using logistic regression and pooling according to Rubin's rule [11]. Analyses were conducted using SAS Enterprise Guide version 8.3.

## 3 Results

**FIGURE 1:**
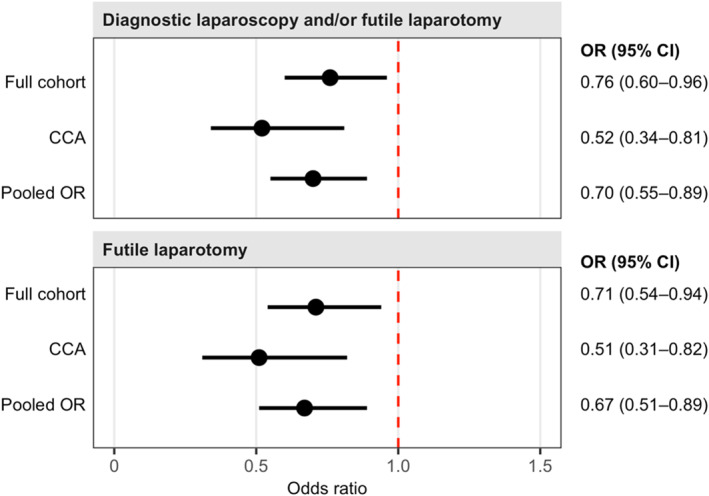
Odds ratios for laparotomy and/or laparoscopy comparing presence versus absence of preoperative abdominal MRI. Odds ratios are comparing participants who did versus did not (reference) undergo a preoperative abdominal MRI. Full cohort includes those with missing/unknown stage; CCA excludes those with missing/unknown stage; pooled ORs resulted from aggregating effect estimates across 50 imputed datasets according to Rubin's rule. OR < 1 favors preoperative abdominal MRI. CCA = complete case analysis, CI = confidence interval, MRI = magnetic resonance imaging, OR = odds ratio.

A total of 4032 patients were included (mean age: 67.0 years [SD = 9.8]), including 2022 (50.1%) who underwent preoperative abdominal MRI (Table 1). Patients who underwent MRI were younger than those who did not (mean age: 66.2 vs. 67.8 years, STD = 0.16), more likely to have received neoadjuvant radiation (5.1% vs. 3.1%, STD = 0.10) and chemotherapy (21.1% vs. 14.9%, STD = 0.16), more likely to have stage I disease (12.6% vs. 7.4%, STD = 0.17), and less likely to have unstaged disease (36.4% vs. 44.5%, STD = 0.17). The MRI group also had a longer interval from diagnosis to surgery (mean: 71.2 vs. 50.2 days, STD = 0.27), whereas differences in sex, income quintile, geographic remoteness, and comorbidity were not meaningfully different. Overall, 324 patients (8.0%) underwent diagnostic staging laparoscopy and/or futile laparotomy, including 130 (6.4%) in the MRI group and 194 (9.7%) in the non‐MRI group; futile laparotomy occurred in 92 (4.5%) and 143 (7.1%) patients, respectively. After propensity score weighting, preoperative abdominal MRI was associated with lower odds of diagnostic staging laparoscopy and/or futile laparotomy (OR = 0.76, 95% CI: 0.60–0.96), with similar results across sensitivity analyses and when futile laparotomy was examined separately (Figure 1).

**TABLE 1:** Patient characteristics by receipt of preoperative abdominal MRI.

Variable	Did not receive MRI (*n* = 2010)	Received MRI (*n* = 2022)	Standardized Difference (STD)
Baseline			
Female sex, *n* (%)	941 (46.8%)	972 (48.1%)	0.025
Age in years			
Mean (SD)	67.81 (9.65)	66.24 (9.94)	0.16
Median (IQR)	69 (14)	67 (14)	0.155
Neighborhood income quintile, *n* (%)			
Q1 (lowest)	342 (17.0%)	351 (17.4%)	0.009
Q2	430 (21.4%)	417 (20.6%)	0.019
Q3	385 (19.2%)	416 (20.6%)	0.036
Q4	391 (19.5%)	394 (19.5%)	0.001
Q5 (highest)	462 (23.0%)	444 (22.0%)	0.025
Geographic remoteness (0–1)			
Mean (SD)	0.09 (0.09)	0.09 (0.09)	0.022
Median (IQR)	0 (0)	0 (0)	0.003
Comorbidity index, *n* (%)			
Charlson = 0	681 (33.9%)	760 (37.6%)	0.077
Charlson = 1	194 (9.7%)	193 (9.5%)	0.004
Charlson = 2	130 (6.5%)	164 (8.1%)	0.063
Charlson> = 3	91 (4.5%)	87 (4.3%)	0.011
No hospital admissions	914 (45.5%)	818 (40.5%)	0.101
Neoadjuvant treatment, *n* (%)			
Radiation	62 (3.1%)	103 (5.1%)	0.102
Chemotherapy	299 (14.9%)	427 (21.1%)	0.163
Cancer stage, *n* (%)			
I	148 (7.4%)	254 (12.6%)	0.174
II	685 (34.1%)	706 (34.9%)	0.018
III	282 (14.0%)	325 (16.1%)	0.057
Unknown/not staged	895 (44.5%)	737 (36.4%)	0.165
Time elapsed from diagnosis to surgery, in days			
Mean (STD)	50.24 (68.43)	71.21 (84.45)	0.273
Median (IQR)	29 (53)	40 (81)	0.268
Outcomes			
Diagnostic laparoscopy and/or futile laparotomy, *n* (%)	194 (9.7%)	130 (6.4%)	0.119
Futile laparotomy, *n* (%)	143 (7.1%)	92 (4.5%)	0.11

*Note:* Standardized differences (STD) > 0.1 considered a statistically meaningful difference.

Abbreviations: IQR = interquartile range, SD = standard deviation.

## 4 Discussion

In this population‐based retrospective study, patients with pancreatic adenocarcinoma who underwent preoperative abdominal MRI had a lower likelihood of futile laparotomy or diagnostic staging laparoscopy. Similar findings were observed when futile laparotomy was evaluated independently, enhancing clinical interpretability of our findings. These results are clinically plausible given that MRI, relative to CT, exhibited improved detection of occult liver metastases [5].

The OCR enabled near‐complete case ascertainment of eligible cases across the province, however, this study has several limitations. First, this was an observational analysis and should not be interpreted as demonstrating a causal effect. Second, given the nature of administrative data, we could not determine indications for MRI, imaging findings or protocols, multidisciplinary decision‐making, or intraoperative factors that changed management. Although indications could not be ascertained, it is plausible that MRI was preferentially used in patients with higher‐risk features or with indeterminate liver lesions on CT. Third, cancer stage was missing for a substantial proportion of patients; however, consistency of findings across analyses supports the robustness of our conclusions.

Future randomized trials or prospective observational studies incorporating detailed clinical and radiologic data are needed to further evaluate the utility of MRI as an add‐on or an alternative to CT in determining resectability in pancreatic adenocarcinoma. Economic evaluations should also be undertaken. Moreover, comprehensive geospatial analyses could help clarify how MRI access modifies care pathways and outcomes for pancreatic cancer patients living in rural and sparsely populated regions.

## Author Contributions

**Joshua O. Cerasuolo:** conceptualization, investigation, writing – original draft, writing – review and editing, visualization, validation, methodology, software, formal analysis, project administration, resources, data curation. **Pablo E. Serrano:** conceptualization, investigation, writing – review and editing, visualization, methodology, validation, supervision. **Noori Akhtar‐Danesh:** conceptualization, investigation, methodology, validation, visualization, writing – review and editing. **Amer Alaref:** conceptualization, investigation, methodology, validation, visualization, writing – review and editing, funding acquisition. **David W. Savage:** conceptualization, investigation, methodology, validation, visualization, writing – review and editing. **Joseph M. Caswell:** conceptualization, investigation, methodology, validation, visualization, writing – review and editing. **Christian B. van der Pol:** conceptualization, investigation, funding acquisition, methodology, validation, visualization, writing – review and editing, supervision.

## Conflicts of Interest

The authors declare no conflicts of interest.

## Data Availability

The dataset from this study is held securely in coded form at ICES. While legal data sharing agreements between ICES and data providers (e.g., healthcare organizations and government) prohibit ICES from making the dataset publicly available, access may be granted to those who meet pre‐specified criteria for confidential access, available at www.ices.on.ca/das (email: das@ices.on.ca). The underlying analytic code is available from authors upon request, understanding that the computer programs may rely upon coding templates or macros that are unique to ICES and are, therefore, either inaccessible or may require modification.
